# Genetic Loss of LCK Kinase Leads to Acceleration of Chronic Lymphocytic Leukemia

**DOI:** 10.3389/fimmu.2020.01995

**Published:** 2020-09-02

**Authors:** Melanie Märklin, Alexander R. Fuchs, Claudia Tandler, Jonas S. Heitmann, Helmut R. Salih, Joseph Kauer, Leticia Quintanilla-Martinez, Stefan Wirths, Hans-Georg Kopp, Martin R. Müller

**Affiliations:** ^1^Department of Hematology, Oncology and Clinical Immunology and Rheumatology, University of Tübingen, Tübingen, Germany; ^2^Clinical Collaboration Unit Translational Immunology, German Cancer Consortium (DKTK), University Hospital Tübingen, Tübingen, Germany; ^3^Department of Immunology, Interfaculty Institute for Cell Biology, University of Tübingen, Tübingen, Germany; ^4^Department of Pathology, University of Tübingen, Tübingen, Germany; ^5^Department of Molecular Oncology and Thoracic Oncology, Robert-Bosch-Hospital Stuttgart, Stuttgart, Germany; ^6^Department of Hematology, Oncology and Immunology, Klinikum Region Hannover, KRH Klinikum Siloah, Hanover, Germany

**Keywords:** CLL, NFAT2, NFATc1, LCK, Richter's transformation, anergy, BCR

## Abstract

Most patients with chronic lymphocytic leukemia (CLL) exhibit an indolent disease course and unresponsive B cell receptors (BCRs) exemplified by an anergic phenotype of their leukemic cells. In up to 5% of patients, CLL transforms from an indolent subtype to an aggressive form of B cell lymphoma (Richter's syndrome), which is associated with worse disease outcome and severe downregulation of NFAT2. Here we show that ablation of the tyrosine kinase LCK, which has previously been characterized as a main NFAT2 target gene in CLL, leads to loss of the anergic phenotype, thereby restoring BCR signaling, which results in an acceleration of CLL. Our study identifies LCK as a main player in mediating BCR unresponsiveness and its role as a crucial regulator of anergy in CLL.

## Introduction

Chronic lymphocytic leukemia (CLL) is characterized by a clonal expansion of mature CD5^+^CD19^+^ B cells and constitutes the most common leukemia in adults. By the introduction of monoclonal antibodies targeting CD20 (rituximab, obinutuzumab and ofatumumab) (1, 2) and small molecule inhibitors (ibrutinib, idelalisib & venetoclax) (3–5) treatment options have been substantially improved. Nevertheless, CLL is still considered an incurable disease. While the majority of patients exhibit an indolent disease course and do not require therapy for many years, other patients show aggressive disease phenotype with early progression and requirement for therapy. A severe progression of the disease is termed Richter's syndrome, which constitutes a transformation to an aggressive lymphoma and occurs in about 5% of CLL patients (6).

Several prognostic factors like unmutated immunoglobulin heavy chain (IGHV) gene loci, high expression of ZAP70 and CD38 as well as certain cytogenetic abnormalities (e.g., del17p) have been associated with dismal treatment outcome (7). Progression of the disease was also shown to be highly dependent on the signaling capacity of the B cell receptor (BCR) [for review (8, 9)]. Previous studies demonstrated a strong tendency for indolent CLL cases to exhibit anergic features like surface IgM (sIgM) downregulation and reduced BCR signaling, in line with elevated basal intracellular Ca^2+^ and constitutive ERK1/2 phosphorylation (10–13). NFAT2 (NFATc1) is known as an important Ca^2+^ dependent transcription factor in lymphocyte development [for review (14)] and has been shown to be overexpressed and constitutively activated in a subset of CLL patients (15, 16). We and others further reported that indolent CLL cells show a high degree of NFAT2 expression correlating with non-responsive, i.e., anergic BCRs (15, 16).

Using the *E*μ*-TCL1* mouse model, which is the most widely used animal model to study pathophysiology, clonal evolution and drug sensitivity in CLL (17–19), we could characterize that CD5^+^CD19^+^ leukemic cells from Eμ-TCL1 transgenic mice possess many important features of anergic CLL cells. Although this model has limitations, we demonstrated that CD5^+^CD19^+^ leukemic cells showed impaired Ca^2+^ mobilization capacity as well as a reduced expression level of sIgM when compared to physiological B cells. We further reported that these cells exhibit a constitutive activation of NFAT2 and ERK1/2 (16). We also recently demonstrated that loss of NFAT2 in the B cell compartment causes an aggressive course of CLL, enhances BCR signaling and results in the selection of unmutated BCRs and leads to a highly proliferative disease in a murine *E*μ*-TCL1* leukemia model (16, 20). This was mirrored by low NFAT2 expression in patients with Richter transformation (16). Since anergy is a hallmark of CLL (9, 21), we established a gene signature contributing to the anergic state of CLL cells, which includes *Cbl-b, Grail, Egr2* and the tyrosine kinase *Lck*. Chromatin-immunoprecipitation studies in primary human CLL cells further resulted in the first report of *Lck* as a NFAT2 target gene in CLL (16, 22).

The LCK kinase, originally discovered in T cells (23), was found to be expressed in both physiological and malignant B cells (24–26). In physiological B cells, LCK has been shown to display opposing functions by potentiating or suppressing BCR signaling (27, 28), while in CLL patients, LCK expression identifies a subpopulation with aberrant BCR signaling (29). Several previous studies did not detect a correlation between LCK expression and clinical outcome (30–32). Overall, the role of LCK in CLL is still not completely understood. Our previous finding that *Lck* is a direct target gene of NFAT2 in CLL and the essential role of LCK in acceleration and transformation of CLL (16, 20, 22) prompted us to investigate its precise function in disease pathophysiology. To this end, we employed a combination of the *E*μ*-TCL1* transgenic mouse model and the LCK knockout model to further demonstrate that LCK deletion leads to acceleration and reversal of the anergic phenotype of CLL. In summary our data confirm that LCK plays a major role in maintaining the anergic phenotype in CLL.

## Materials and Methods

### Mice

*NFAT2*^fl/fl^ *CD19-Cre* (NFAT2-KO) mice and transgenic *E*μ*-TCL1* mice [kindly provided by C. M. Croce (33)] have been described previously (16). B6.129S2-*Lck*^*tm1Mak*^/J (LCK-KO) mice were kindly provided by L. Simeoni from Magdeburg, Germany. Mice were used on the C57BL/6 background (CRL 027) and maintained under specific pathogen-free conditions. Homozygous *TCL1 NFAT2*^*fl*/^^fl^ *CD19*^*Cre*^ (TCL1 NFAT2-KO*)* mice with B cell-specific deletion of NFAT2 and *TCL1 NFAT2*^*fl*/^^fl^ (TCL1) mice without NFAT2 deletion served as controls. Both cohorts were crossed with the LCK-KO strain to obtain the experimental cohorts exhibiting a germline deletion of LCK kinase. All mice were age and sex-matched and were sacrificed at the indicated time points by overdosing inhalation anesthesia. Mice exhibiting clinical signs of disease or >20% weight loss were removed early from the experimental cohort. Animal experiments were performed with the authorization of the Institutional Animal Care and Use Committee of the University of Tübingen according to German federal and state regulations following the ARRIVE guidelines (34).

### Flow Cytometry

Cell suspensions of different organs were prepared as previously described (16) and stained with fluorophore labeled mAbs. For detailed information see Supplementary Material.

### Ca^2+^ Measurements

Splenic B cells were freshly isolated by density gradient centrifugation and stained as previously described (16). In brief, cells were stained with CD19-FITC and CD5-APC mAb for 20 min, washed and loaded with 10 μg/ml FuraRed and 0.02% Pluronic F127 (both from Thermo Fisher) for 25 min at 30°C. Baseline was recorded in 4 mM Ca^2+^ Krebs-Ringer solution for 30 s and Ca^2+^ mobilization was assessed after stimulation of the cells with 10 μg/mL α-IgM F(ab′)_2_ (Jackson ImmunoResearch). After 3 min of recording, 1 μM Ionomycin was added as a positive control. Increases in free intracellular Ca^2+^ were measured in real-time with the Canto II cytometer. To determine the Ca^2+^ flux, the ratio of bound and unbound FuraRed was calculated with the FlowJo software.

### Phospho Flow Analysis

Splenic B cells were stained with CD19-BV510 and CD5-APC mAb for 15 min, washed and diluted in FCS. Cells were stimulated with 10 μg/mL α-IgM (Southern Biotech) for the indicated time points at 37°C. Cells were immediately fixed and permeabilized with the PerFix EXPOSE Kit (Beckman Coulter) according to the manufacturer's instructions. Intracellular staining of ERK1/2/P-ERK1/2(T202/Y204) (1:400/1:800) and SYK/P-SYK(Y525/526) (both 1:200) and respective isotype controls was performed, followed by detection with an α-rabbit F(ab′)_2_-PE conjugate (1:250) (all from Cell Signaling Technology) and measured with the LSRFortessa cytometer (BD Bioscience).

### Real-Time RT-PCR

RNA isolation and qRT-PCR was performed as previously described (16). Primer sequences were shown in Supplementary Table 1.

### Western Blot

Western blotting of isolated B cells was performed with a LI-COR Odyssey imaging system as previously described (16). Primary antibodies for LCK (#2752, 1:1,000) and Cofilin (D3F9, 1:2,000) (both from Cell signaling) were used for detection.

### Immunohistochemistry

Spleens were fixed in 4% formalin, embedded in paraffin and immunohistochemistry and microscopy was performed as previously described (16).

### Statistical Analysis

For statistical analysis GraphPad Prism 8 was used. Mean values and standard error of the mean (SEM) are shown. The 95% confidence level was used and *p*-values were calculated with an unpaired two-tailed Student's *t*-test in the case of normally distributed data. Significance of not normally distributed data was calculated with a paired two-tailed Wilcoxon matched-pairs signed-rank test. An unpaired analysis of variance (ANOVA) was used to analyze the differences among group means. Significance of survival data was calculated by using a Log-rank (Mantel-Cox) test. Significant *p*-values *p* < 0.05 were marked with ^*^.

## Results and Discussion

**Figure 1:**
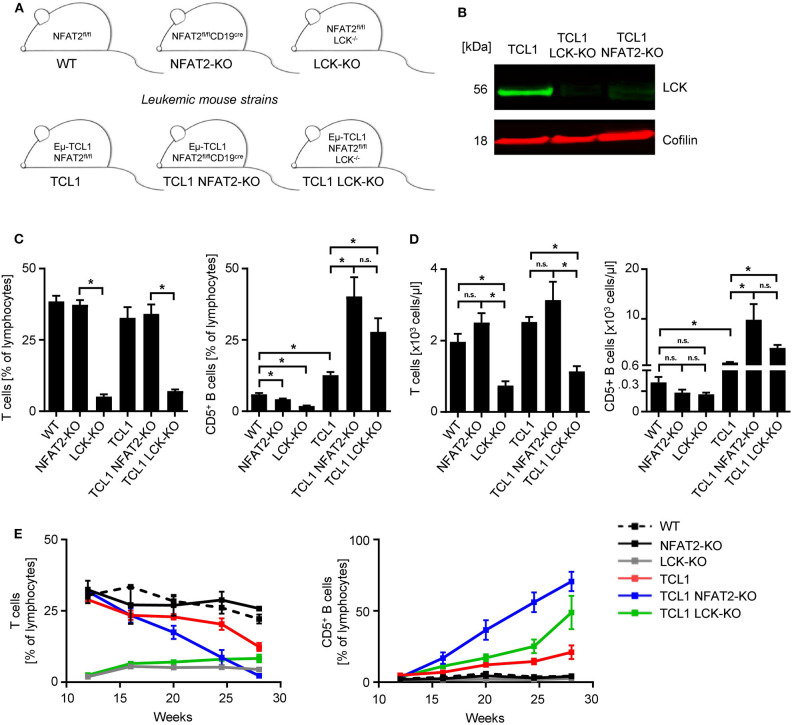
LCK deletion leads to increased CD5^+^ B cells in TCL1 leukemic mice. **(A)** Mouse strain schemes of all mice utilized in this study are depicted. **(B)** CD19^+^ splenic B cells from TCL1, TCL1 LCK-KO, and TCL1 NFAT2-KO mice were isolated and LCK protein expression was assessed by western blotting. Cofilin was used as loading control. One exemplary result out of three is shown. **(C)** Peripheral blood of mice with the indicated genotype at the age of 23 weeks was analyzed for CD3^+^ T cells (left) and CD5^+^ B cells (right) by flow cytometry (CD3^+^ T: WT/NFAT2-KO/TCL1/TCL1 NFAT2-KO *n* = 5; LCK-KO *n* = 8; TCL1 LCK-KO *n* = 10 per group; CD5^+^ B cells: TCL1 LCK-KO *n* = 6; LCK-KO *n* = 7; WT *n* = 10; NFAT2-KO *n* = 9; TCL1/TCL1 NFAT2-KO *n* = 8 per group). **(D)** Absolut numbers of CD3^+^ T cells (left) and CD5^+^ B cells (right) in the peripheral blood of mice with the indicated genotype at the age of 23 weeks (CD3^+^ T: NFAT2-KO/LCK-KO/TCL1 *n* = 8; WT/TCL NFAT2-KO *n* = 9; TCL1 LCK-KO *n* = 10 per group; CD5^+^ B cells: WT/NFAT2-KO/LCK-KO/TCL1 LCK-KO *n* = 7; TCL1 *n* = 6; TCL1 NFAT2-KO *n* = 9 per group). **(E)** Expansion of CD3^+^ T cells and CD5^+^ B cells in the peripheral blood of mice with the respective genotypes was assessed at the indicated time points (WT/NFAT2-KO *n* = 5; LCK-KO *n* = 9; TCL1/TCL1 LCK-KO *n* = 11; TCL1 NFAT2-KO *n* = 10 per group at the beginning of the observation). Multiple comparisons of the different groups at week 28 is shown in Supplementary Table 2. n.s., not significant; *p < 0.05.

To analyze how the genetic loss (KO) of LCK impacts disease progression, we bred a conditional NFAT2-KO strain expressing the TCL1 oncogene with germline LCK-KO mice (Figure 1A). LCK deletion in B cells was confirmed by Western blot analysis (Figure 1B). We analyzed a new cohort of the previously published TCL1 and the TCL1 NFAT2-KO mice in comparison with the newly generated TCL1 LCK-KO mice in a new set of experiments.

As it was already known that LCK-KO mice show compromised T cell compartments, we first analyzed the T cells of different KO strains. As expected, LCK deletion leads to a severe reduction of T cells, independent of leukemia induction in the TCL1 expressing cohort (Figures 1C,D). NFAT2 deletion also leads to decreased CD5^+^ B cell populations compared to WT mice with intact NFAT2 expression. LCK-KO mice show a further decreased CD5^+^ B cell subset, indicating an essential role for both NFAT2 and LCK in CD5^+^ B cell development. Interestingly, while TCL1 NFAT2-KO mice exhibit an increased CD5^+^ B cell population compared to TCL1 mice as described before (16), TCL1 LCK-KO mice show a similarly expanded CD5^+^ B cell compartment, comparable to the TCL1 NFAT2-KO mice (Figures 1C,D). This suggests that knockout of LCK can, at least in part, substitute for the deletion NFAT2, thereby emphasizing the essential role of LCK as a main player in CLL.

Analyses over an extended time period of 28 weeks confirmed these data and again demonstrated that T cell compartments are virtually not affected during leukemia development, but as expected decreased exclusively due to CD5^+^ B cell expansion in the different mouse cohorts (Figure 1E, Supplementary Table 2). A slightly lower expansion of CD5^+^ B cells in TCL1 LCK-KO mice compared to TCL1 NFAT2-KO mice indicates that LCK is not the exclusive player in CLL acceleration. However, it should be highlighted that in contrast to deleting a multipotent transcription factor like NFAT2, knockout of a single kinase mimics the respective phenotype.

**Figure 2:**
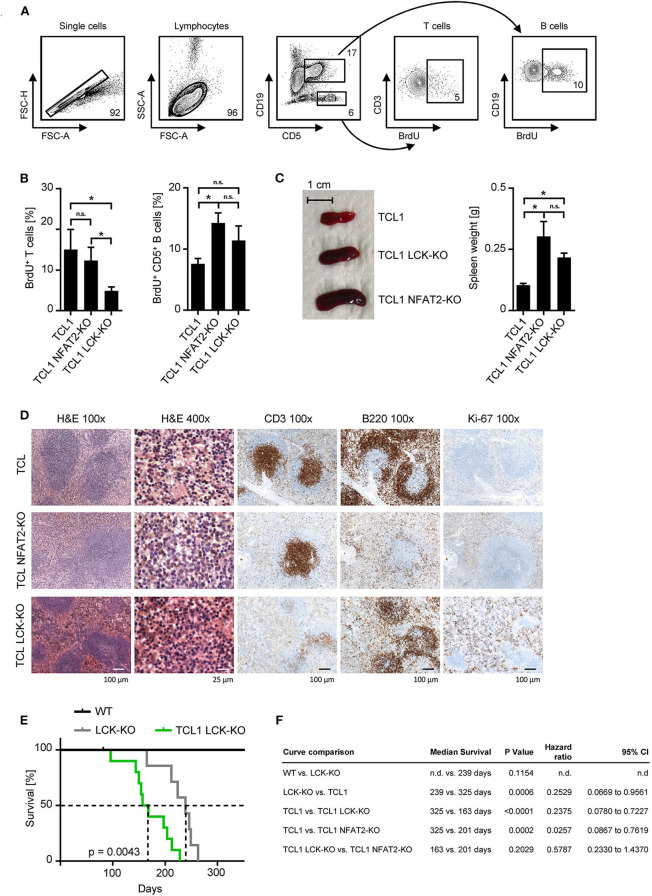
LCK deletion leads to acceleration of leukemia and decreased survival of TCL1 transgenic mice. **(A)** Proliferation of CD3^+^ T cells and CD5^+^ B cells in the peripheral blood was assessed. Mice were injected with 10 mM BrdU i.p. and peripheral blood cells were harvested after 48 h. Cells were stained with αBrdU and measured by flow cytometry. One exemplary result of the TCL1 LCK-KO group with gating strategy is shown. **(B)** Pooled data of the BrdU analysis for the indicated genotypes at the age of 20 weeks (*n* = 5 per group). **(C)** Spleen sizes of representative animals of the indicated genotypes at an age of 25 weeks are shown and average spleen weight of TCL1 (*n* = 9), TCL1 NFAT2-KO (*n* = 8), and TCL1 LCK-KO (*n* = 6), mice at the age of 25 weeks is shown. **(D)** H & E staining of paraffin-embedded spleen sections of one representative TCL1, TCL1 LCK-KO, and TCL1 NFAT2-KO mouse out of three at an age of 25 weeks (left panels) and immunohistochemical staining for CD3, B220 and Ki-67 (right panels). **(E)** Kaplan-Meier survival plot of WT (*n* = 7), LCK-KO (*n* = 7) and TCL1 LCK-KO (*n* = 10) mice is depicted. Median survival is indicated with dashed lines. **(F)** Log-rank test of the Kaplan-Meier analysis of WT, LCK-KO and TCL1 LCK-KO mice in the present study compared to the previous published TCL1 and TCL1 NFAT2-KO cohorts (16) with median survival, hazard ratio and the 95% confidence interval (CI) is shown. (n.d., not determined). n.s., not significant; *p < 0.05.

Further characterization of the underlying mechanism showed that *in vivo* proliferation, as assessed by BrdU incorporation was diminished in LCK-deficient T cells in the leukemic cohorts (Figures 2A,B). CD5^+^ B cells of TCL1 NFAT2-KO and TCL1 LCK-KO cohorts showed significantly higher proliferation rates compared to the TCL1 group, pointing to a differing role for LCK in T and B cells. The observed B cell expansion was in line with the increased spleen size of TCL1 mice with NFAT2 or LCK KO compared to TCL1 mice with intact expression of these genes (Figure 2C). Immunohistochemistry analyses in the TCL1 group showed a normal architecture of the spleen with mild expansion of the white pulp, while the red pulp showed a subtle lymphocytic infiltrate of B cells (B220), with mature chromatin without nucleoli and low mitotic activity as demonstrated by Ki-67 staining (Figure 2D). In contrast, spleens in the TCL1 NFAT2-KO group showed mild to severe atrophy of the white pulp and infiltration of B cells with mature chromatin, conspicuous nucleoli and scant cytoplasm in the red pulp. Ki-67 staining revealed a higher proliferation rate in the spleens compared to the TCL1 group. The TCL1 LCK-KO group showed atrophy of the white pulp and expansion of the red pulp, which was infiltrated by small size B lymphocytes (B220). Of note, decreased B220 expression in NFAT2 deficient mice was already shown before (16) and B cell identity was shown with additional staining of CD79a (Supplementary Figure 1). The infiltrating small lymphoid cells displayed scant cytoplasm and mature chromatin. CD3 staining revealed the atrophy of the white pulp. In both KO cohorts, the proliferation rate was very high as revealed by Ki-67 immunohistochemistry. Of note, Ki-67 staining in the TCL1 LCK-KO group was additionally seen in the red pulp reflecting reactive hematopoiesis. Taken together, we observed comparable malignant infiltration and disruption of the spleen architecture in both knockout cohorts, which provides further evidence for the significance of LCK as disease-modifying factor in CLL.

The combined data observed with peripheral blood and spleens clearly demonstrate the acceleration of CLL due to the genetic loss of LCK kinase. Prompted by our previously published data (16) we performed long term survival analyses with the LCK deficient mouse cohorts (Figure 2E). Compared to healthy WT mice LCK-KO control mice showed a significantly reduced median life span during the observation period of 350 days, due to their lack of T cells and the resulting immunodeficiency (239 days vs. not reached). For this reason, TCL1 mice with LCK deletion were thoroughly monitored and mice which died from infection instead of leukemic progression were excluded from the analysis. Comprehensive statistical analyses of TCL1 and TCL1 NFAT2-KO mice obtained in the previous study (16) and the newly generated LCK deficient cohorts revealed no significant difference regarding the median survival of TCL1 mice with NFAT2 deletion compared to TCL1 mice with LCK deletion (201 vs. 163 days), whereas TCL1 mice with intact LCK expression survived significantly longer than TCL1 LCK-KO mice (325 vs. 163 days) (Figure 2F). However, we are aware of the confounded effects of the germline LCK-KO, which could be avoided by adoptive transfer experiments in the future. Nevertheless, these data implies that loss of LCK leads to decreased survival of leukemic animals, which is most likely due to increased cell proliferation and acceleration of CLL progression.

**Figure 3:**
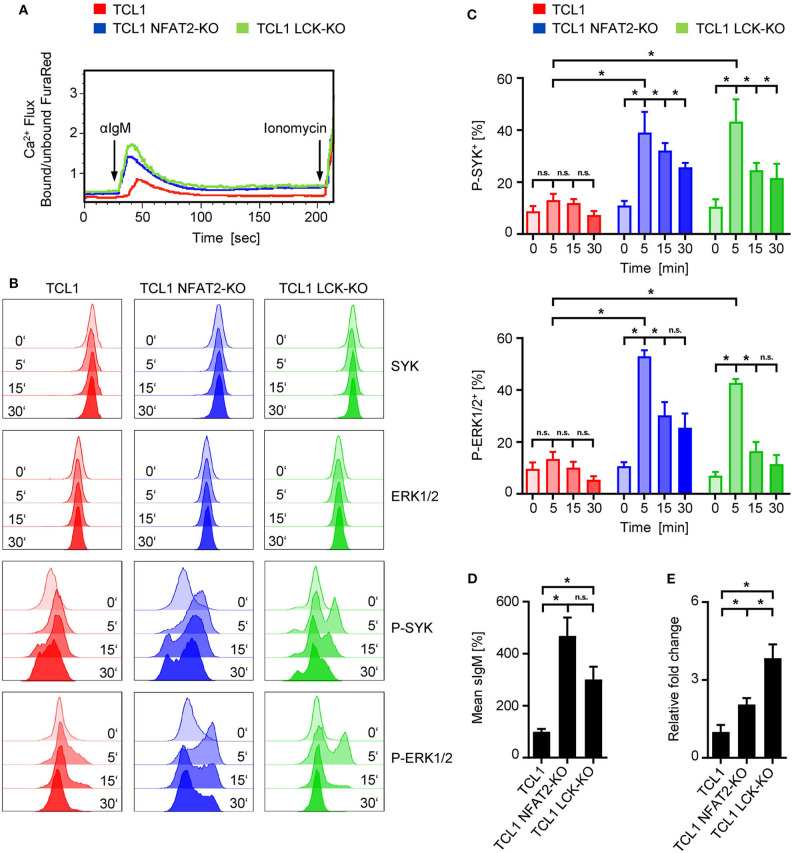
Loss of LCK restores BCR signaling in TCL1 transgenic mice. **(A)** Ca^2+^ flux analysis of splenic CD5^+^ B cells from TCL1, TCL1 NFAT2-KO, and TCL1 LCK-KO mice at an age of ~18 weeks after stimulation with 10 μg/mL αIgM is depicted. Ionomycin (1 mM) was added as positive control. One representative result of three experiments is shown. **(B,C)** After staining of splenocytes (~20 weeks old mice) for CD19 and CD5, cells were stimulated with 10 μg/mL αIgM for the indicated time points and BCR activation was assessed by intracellular staining of SYK/P-SYK(Y525/526) and ERK1/2/P-ERK1/2(T202/Y204) in CD5^+^ B cells with flow cytometry. **(B)** One representative result out of four independent experiments is shown. **(C)** Pooled result of P-SYK^+^ and P-ERK1/2^+^ CD5^+^ B cells at the indicated time points of TCL1, TCL-NFAT2-KO and TCL LCK-KO mice (TCL1 *n* = 6, TCL1 NFAT2-KO/TCL1 LCK-KO *n* = 5) is shown. **(D)** IgM surface expression on B cells from TCL1, TCL1 NFAT2-KO, and TCL1 LCK-KO mice was determined by flow cytometry (TCL1/TCL1 LCK-KO *n* = 8, TCL1 NFAT2-KO *n* = 7 per group, ~18 weeks old). Mean expression levels of IgM were normalized to TCL1 mice. One representative result of four experiments is shown. **(E)** Relative gene expression in splenic B cells of *Prdm1* in TCL1 NFAT2-KO and TCL1 LCK-KO was normalized to mice normalized TCL1 mice. Relative gene expression was calculated by ratio of *Prdm1* to *Actin* expression from (TCL1/TCL1 NFAT2-KO *n* = 5, TCL1 LCK-KO *n* = 4 per group, ~18 weeks old). n.s., not significant; *p < 0.05.

Differential B cell receptor (BCR) signaling was hypothesized as potential mechanism underlying these observations, since LCK is an early kinase proximal to the BCR and is strongly active in TCL1 mice exhibiting a more indolent disease course (16, 32). To test whether the BCR of TCL1 LCK-KO also displayed a hyperresponsive phenotype, as we have previously observed for TCL1 NFAT2-KO mice, we performed Ca^2+^ mobilization analyses (Figure 3A). Stimulation of the BCR with an anti-IgM antibody revealed a significantly decreased Ca^2+^ flux capacity of TCL1 mice, which were previously characterized to exhibit an anergic phenotype (16), whereas TCL1 mice lacking LCK expression showed increased Ca^2+^ mobilization comparable to TCL1 NFAT2-KO mice. To further elucidate the downstream signaling of the BCR, SYK and ERK1/2 activation were analyzed in splenocytes by phosphorylation flow cytometry. Stimulation with an anti-IgM antibody showed induction of P-SYK and P-ERK1/2 in CD5^+^ B cells of TCL1 NFAT2-KO and TCL1 LCK-KO mice over time, while total protein levels were not affected. In contrast, TCL1 mice exhibit a significantly less responsive BCR which results in just very weak induction of P-SYK and P-ERK1/2 (Figures 3B,C). Of note, specificity of this assay could be shown by the absence of any P-ERK1/2 induction in T cells contained in the splenocyte population (Supplementary Figure 2).

Since it was previously described that sIgM levels directly correspond with BCR responsiveness (12, 35–37), we analyzed IgM surface expression levels (Figure 3D). Here, we could observe a similar upregulation of sIgM in LCK deficient TCL1 B cells as it has been recently documented for TCL1 NFAT2-KO B cells, thus supporting the concept of hyperresponsive BCRs in aggressive CLL. Anergic CLL cells have also been characterized to exhibit depression of *Prdm1* (*Blimp-1*) expression (16, 38). In LCK deficient TCL1 mice, we observed a substantial increase of *Prdm1* gene expression compared to TCL1 mice (Figure 3E), which supports our data on BCR hyperresponsiveness and aggressive disease acceleration caused by LCK knockout.

In summary, our data clearly support the hypothesis that LCK is a critical regulator of the anergic phenotype in CLL and that its loss leads to hyperresponsive BCRs and disease acceleration in the *E*μ*-TCL1* mouse model.

## Data Availability Statement

The raw data supporting the conclusions of this article will be made available by the authors, without undue reservation.

## Ethics Statement

The animal study was reviewed and approved by Institutional Animal Care and Use Committee of the University of Tübingen. Written informed consent was obtained from the owners for the participation of their animals in this study.

## Author Contributions

MM, AF, JH, CT, SW, LQ-M, and MMü designed and performed the experiments. MM, JH, JK, LQ-M. and MMü analyzed data and wrote the manuscript. MRM, HS, and H-GK designed research, analyzed data, and provided important advice. All authors contributed to the article and approved the submitted version.

## Conflict of Interest

The authors declare that the research was conducted in the absence of any commercial or financial relationships that could be construed as a potential conflict of interest.

## Abbreviations

BCR: B cell receptor
CLL: chronic lymphocytic leukemia
IGHV: immunoglobulin heavy chain
NFAT: nuclear factor of activated T cells
SEM: standard error of the mean.
