# Supplementary material for: Genetic Loss of LCK Kinase Leads to Acceleration of Chronic Lymphocytic Leukemia
Source: Front Immunol. 2020 Sep 2;11:1995. doi: 10.3389/fimmu.2020.01995 (PMC7492521; doi:10.3389/fimmu.2020.01995)
Supplement: Supplementary file 1 [file Data_Sheet_1.docx]

Supplementary Material

# Supplementary Table S1

| β-Actin fwd | 5’‑TCTTGGGTATGTAATCCTGTGGCA‑3’ |
| --- | --- |
| β-Actin rv | 5’‑ACTCCTGCTTGCTGATCCACATCT‑3’ |
| Prdm1 fwd | 5’‑TAGACTTCACCGATGAGGGG‑3’ |
| Prdm1 rv | 5’‑GTATGCTGCCAACAACAGCA‑3’ |

**Supplementary Table S1:** Primer sequences for mouse genes.

# Supplementary Methods

**Flow cytometry**

Cell suspensions of different organs were prepared as previously described^13^ and stained with CD19-FITC/APC/PECy7 (eBio1D3, 1:100), IgM-PE/PECy7 (II/41, 1:200), CD5-APC/Pacific Blue (53-7.3, 1:80), CD3-FITC (145-2C11, 1:100), Annexin V-Pacific Blue (1:25) and 7-AAD (1:100) (all from eBioscience). For *in vivo* proliferation analysis the BrdU Flow Kit APC (BD Bioscience) was used according to the manufacturer’s instructions. Cells were analyzed with the Canto II cytometer (BD Bioscience). Data was obtained with the BD FACSDiva^TM^ (BD Bioscience) and analyzed with the FlowJo_V10 software.

|  | **T cells** | | | | | **CD5^+^ B cells** | | | | |
| --- | --- | --- | --- | --- | --- | --- | --- | --- | --- | --- |
|  | **12 weeks** | **16 weeks** | **20 weeks** | **25 weeks** | **28 weeks** | **12 weeks** | **16 weeks** | **20 weeks** | **25 weeks** | **28 weeks** |
|  |  |  |  |  |  |  |  |  |  |  |
| WT vs. NFAT2-KO | 0,9531 | 0,5019 | 0,6558 | 0,6898 | 0.1003 | 0,9810 | 0,9504 | 0,9221 | 0,9837 | 0.9904 |
| WT vs. LCK-KO | <0,0001* | <0,0001* | <0,0001* | <0,0001* | <0.0001* | 0,9810 | 0,9257 | 0,8893 | 0,9837 | 0.9904 |
| WT vs. TCL1 | 0,9598 | 0,0795 | 0,2208 | 0,3048 | <0.0001* | 0,0307* | 0,8042 | 0,8634 | 0,2885 | 0.0407* |
| WT vs. TCL1 NFAT2-KO | 0,9598 | 0,0669 | 0,0014* | <0,0001* | <0.0001* | 0,6727 | 0,0056* | 0,0001* | <0,0001* | <0.0001* |
| WT vs. TCL1 LCK-KO | <0,0001* | <0,0001* | <0,0001* | <0,0001* | <0.0001* | 0,6259 | 0,2094 | 0,3892 | 0,0069* | <0.0001* |
| NFAT2-KO vs. LCK-KO | <0,0001* | <0,0001* | <0,0001* | <0,0001* | <0.0001* | 0,9810 | 0,9504 | 0,9221 | 0,9837 | 0.9904 |
| NFAT2-KO vs. TCL1 | 0,5994 | 0,7510 | 0,4149 | 0,0548 | <0.0001* | 0,0129* | 0,6421 | 0,8244 | 0,2885 | 0.0407* |
| NFAT2-KO vs. TCL1 NFAT2-KO | 0,9598 | 0,7510 | 0,0058* | <0,0001* | <0.0001* | 0,5836 | 0,0010* | 0,0001* | <0,0001* | <0.0001* |
| NFAT2-KO vs. TCL1 LCK-KO | <0,0001* | <0,0001* | <0,0001* | <0,0001* | <0.0001* | 0,5732 | 0,0698 | 0,3539 | 0,0054* | <0.0001* |
| LCK-KO vs. TCL1 | <0,0001* | <0,0001* | <0,0001* | <0,0001* | <0.0001* | 0,0143* | 0,3874 | 0,4173 | 0,1490 | 0.0071* |
| LCK-KO vs. TCL1 NFAT2-KO | <0,0001* | <0,0001* | <0,0001* | 0,5967 | 0.1729 | 0,5732 | <0,0001* | <0,0001* | <0,0001* | <0.0001* |
| LCK-KO vs. TCL1 LCK-KO | 0,9598 | 0,9204 | 0,6558 | 0,6898 | 0.1003 | 0,5417 | 0,0068* | 0,0408* | 0,0012* | <0.0001* |
| TCL1 vs. TCL1 NFAT2-KO | 0,5994 | 0,9793 | 0,1341 | 0,0013* | <0.0001* | 0,2288 | 0,0184* | 0,0006* | <0,0001* | <0.0001* |
| TCL1 vs. TCL1 LCK-KO | <0,0001* | <0,0001* | <0,0001* | 0,0014* | 0.1003 | 0,6259 | 0,5747 | 0,8634 | 0,2885 | 0.0034* |
| TCL1 NFAT2-KO vs. TCL1 LCK-KO | <0,0001* | <0,0001* | 0,0002* | 0,8338 | 0.0146* | 0,9810 | 0,2584 | 0,0023* | <0,0001* | 0.0710 |
|  |  |  |  |  |  |  |  |  |  |  |
| *Results of Figure 1E calculated with an ordinary one-way ANOVA with Holm-Sidak’s multiple comparison of T cells and CD5^+^ B cells over time.* | | | | | | | | | | |

# Supplementary Table S2

**Supplementary Table S2: Statistical analysis of Figure 1E**

Expansion of CD3^+^ T cells and CD5^+^ B cells in the peripheral blood of mice with the respective genotypes was assessed at the indicated time points and the results are shown in Figure 1E. Multiple comparisons of the different groups different time points were calculated with an ordinary one-way ANOVA with Holm-Sidak’s corrections for multiple testing.
