# Supplementary figures and images for: Genetic Loss of LCK Kinase Leads to Acceleration of Chronic Lymphocytic Leukemia
Source: Front Immunol. 2020 Sep 2;11:1995. doi: 10.3389/fimmu.2020.01995 (PMC7492521; doi:10.3389/fimmu.2020.01995)

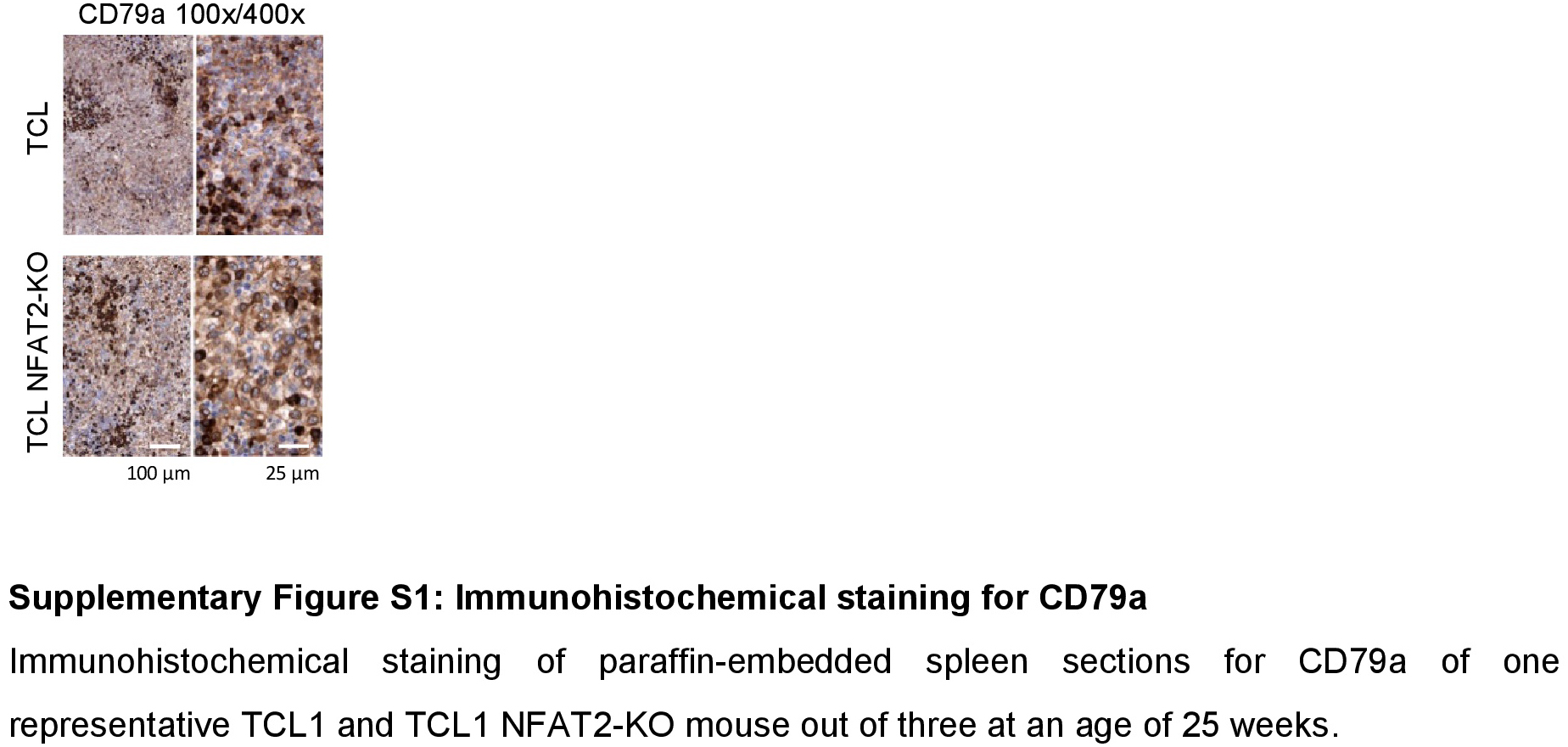

Supplement: Supplementary file 2 [file Image_1.JPEG]

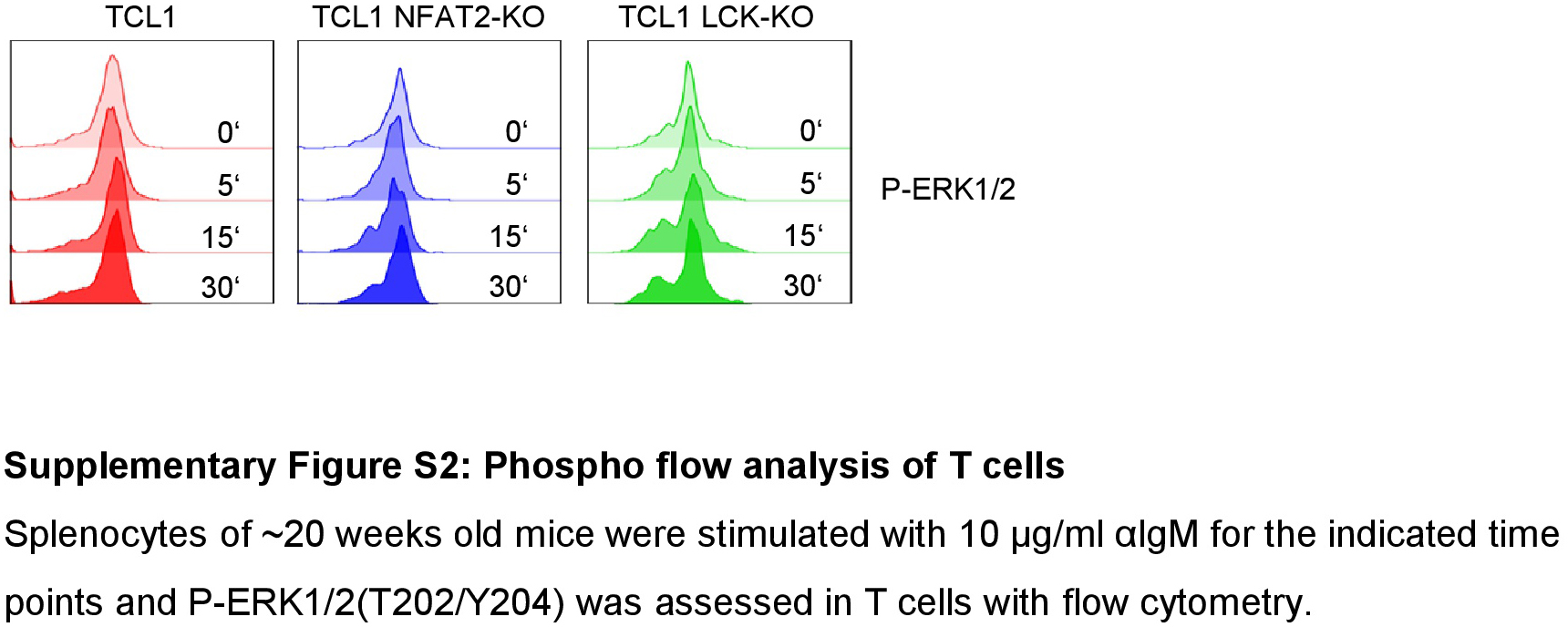

Supplement: Supplementary file 3 [file Image_2.JPEG]
